# Nanofibrous scaffolds in biomedical applications

**DOI:** 10.1186/2055-7124-18-5

**Published:** 2014-06-13

**Authors:** Kailash Chandra Gupta, Adnan Haider, Yu-ri Choi, Inn-kyu Kang

**Affiliations:** Department of Polymer Science and Engineering, Kyungpook National University, Daegu, 702-701 South Korea; Department of Chemistry, Indian Institute of Technology Roorkee, Roorkee, 247 667 India

**Keywords:** Electrospinning, Drug delivery, Wound healing, Tissue engineering, Bioactive agent

## Abstract

Nanofibrous scaffolds are artificial extracellular matrices which provide natural environment for tissue formation. In comparison to other forms of scaffolds, the nanofibrous scaffolds promote cell adhesion, proliferation and differentiation more efficiently due to having high surface to volume ratio. Although scaffolds for tissue engineering have been fabricated by various techniques but electrospun nanofibrous scaffolds have shown great potential in the fields of tissue engineering and regeneration. This review highlights the applications and importance of electrospun nanofibrous scaffolds in various fields of biomedical applications ranging from drug delivery to wound healing. Attempts have also been made to highlights the advantages and disadvantages of nanofirbous scaffolds fabricated for biomedical applications using technique of electrospinning. The role of various factors controlling drug distribution in electrospun nanofibrous scaffolds is also discussed to increase the therapeutic efficiency of nanofibrous scaffolds in wound healing and drug delivery applications.

## Introduction

Nanofibers have played significant role in various fields of biomedical research ranging from drug delivery to wound healing due to having high surface area and opportunities to tune their properties by varying composition and fabrication parameters [1, 2]. The fabrication of electrospun nanofibers using hydrophilic polymers found to be effective in developing fast dissolving delivery systems having reduced drug–drug interactions [3–5]. The nanofibrous scaffolds fabricated with natural and synthetic polymers found to be promising for developing drug delivery systems by electrospinning of blended polymers or through coaxial spinning of two different polymers along with drugs and active agents [6, 7]. The technique of electrospinning has produced nanofibrous scaffolds that not only reduced the bulk release of encapsulated drugs but also found useful in developing dual degree delivery systems for post operative surgical treatment of the patients [8]. Recently a variety of techniques has been evolved to fabricate nanofibrous scaffolds for biomedical applications such as techniques of phase separation [9], self-assembly [10], melt-blowing [11], and templating system [12]. However, the technique of electrospinning found to be useful to produce nanofibrous scaffolds [2, 13, 14] for various biomedical applications. Although nanofibrous scaffolds are potentially useful in various fields but this review focuses on applications of electrospun nanofibrous scaffolds for biomedical applications such as drug delivery [15–17], wound healing [18–25], and delivery of bioactive molecules [26] in tissue engineering.

## Review

This review provides briefly the state of art of elctrospinning for nanofibrous scaffolds and highlights the recent developments in applications of nanofibrous scaffolds in various fields of biomedical research.

## Nanofibrous scaffolds by electrospinning technique

The electrospinning has been known since 1897 based on the principle of Rayleigh [27]. Electrospinning process utilizes the electrostatic forces to draw the fibers from the droplet formed at the tip of spinneret. The applications of electrospinning technique have been explored in various fields [16, 19, 22, 26] and several studies have been conducted to analyze the controlling parameters of electrospinning process [28–38].

**Figure 1:**
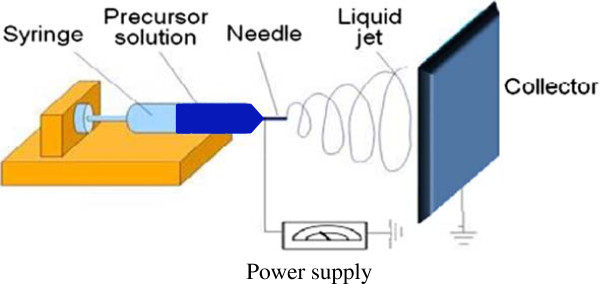
**An electrospinning setup showing the arrangement for syringe, precursor solution, needle, liquid jet, collector, and power supply.**

In principle, the electrospinning needs three basic components such as, a high voltage DC supply, grounded collector, and syringe pump (Figure 1). On application of voltage, charge is induced in the solution and a Taylor cone [36] is formed by the balance of electrical force and surface tension of the solution. After Taylor cone formation, a charged fiber jet is produced, which moves toward grounded collector when applied potential overcomes the surface tension of the solution in Taylor cone. The formation of nanofibers is influenced by various parameters, which are mainly grouped in following three categories (A, B, and C);

A: Solution parameters, which include conductivity, surface tension, and viscosity of solution.
B: Process parameters, which include applied voltage, distance between tip to collector, flow rate, and electric field induced by the collector.
C: Ambient parameters, which include temperature and humidity.

The electrospinning technique is capable of producing continuous fibers using wide range of material such as polymers and their composites with ceramics. It is an effective means to produce non-woven membranes of nanofibers ranging from micron to nanosized diameter [38–42]. The electrospun nanofibers possess high surface to volume ratio, which is responsible for their importance in various applications such as tissue engineering [43–46], drug delivery [47–49], and other bio [50], energy related applications [51–57]. The effect of different surfactants on diameter of poly(lactic acid) (PLA) electrospun fibers has been studied by Zeng et al. [58] and addition of triethylbenzyl ammonium chloride (TEBAC) has significantly reduced the diameter of electrospun nanofibers due to increased conductivity and polarizibility of solution in comparison to solution without surfactant. The addition of non ionic surfactant such as poly(propylene oxide-ethylene oxide) ether (PPO-PEO) (AEO10) has produced uniform fibers, whereas, addition of anionic surfactants such as sodium dodecyl sulfate (SDS) has shown manifold increase in diameter of electrospun nanofibers than electrospun nanofibers produced in presence of cationic and non-ionic surfactants. These investigations have clearly indicated that properties of electrospun nanofibers could be controlled using different additives and solvents.

### Electrospun nanofiberous scaffolds in biomedical applications

Over the last 30 years extensive work has been carried for developing electrospun nanofibrous scaffolds for the promotion of bone tissue formation and to assist in wound healing [18–20]. Recent advancements have led to the development of composite scaffolds, consisting of materials that help in tissue engineering and delivery of drugs [18–21], and growth factors [26]. To control the release profile of encapsulated drugs, various techniques have been applied using polymers of optimal degradation and erosion. The electrospun nanofibrous scaffolds have shown significant control on drug release kinetics and great potential in maintaining therapeutic doses for longer period in comparison to other drug releasing scaffolds.

### Electrospun nanofibrous scaffolds in drug delivery systems

The electrospun nanofibrous scaffolds found to be useful in drug delivery systems [59] due to high surface to volume ratio and high pore connectivity [60]. The polymer based electrospun nanofibrous scaffolds are able to provide useful way out to develop drug delivery systems for a number of recently-developed water insoluble drugs. The electrospun nanofiber technique is also found to be helpful in providing a novel approach for dissolving and releasing of even very poorly soluble drugs. The poly(vinyl pyrrolidone) (PVP) based electrospun fibers found to be potentially useful in improving the solubility of poorly water soluble drugs as PVP based nanofibers are able to provide better dispersal for poorly water soluble drugs [61]. The electrospun nanofibrous scaffolds based drug delivery systems (DDS) have shown great potential in providing better control on drug dosages in comparison to other methods of drug delivery systems [62]. The itraconazole is widely used for the treatment of Tinea Pedis and other fungal infections; hence electrospun nanofibrous scaffolds based drug delivery systems have been developed and analyzed for drug release kinetics [63]. The itraconazole release from electrospun nanofibrous scaffolds has shown linear dependence to the square root of release time, which clearly indicated that itraconazole has followed a Fickian release kinetics. The mefoxin loaded poly(D, L-lactic acid) (PDLLA) electrospun nanofibrous scaffolds have shown complete release of mefoxin within 24 h with a bulk release within initial 3 h due to aggregation of large number of mefoxin molecules at the surface of nanofibrous scaffolds [63]. These investigations have clearly suggested that surface deposition and drug aggregation has significant influence on drug release behavior of electrospun nanofibrous scaffolds. The electrospun nanofibrous scaffolds from poly(ethylene-co-vinyl acetate) (PEVA) and PLA blends containing tetracycline hydrochloride have been used successfully for the treatment of periodontal diseases in comparison to other scaffolds [24]. The PEVA based electrospun nanofibrous scaffolds have shown high release rate than 1:1 ratios of PLA/PEVA or nanofibrous scaffolds obtained from pure PLA. The PEVA based electrospun nanofibrous scaffolds have released 65% of loaded tetracycline hydrochloride within 120 h [64, 65]. The composition, morphology and structures of electrospun nanofibers for drug delivery systems have been controlled easily using emulsion electrospinning techniques [66, 67]. The electrospun nanofibrous scaffolds have also been used successfully in controlled delivery of genes [68], proteins [69–71], and enzymes [72]. The scaffolds having electrospun nanofibers with low diameters was found to be more effective in dual drug delivery [15–17], and applications in post-surgical treatment of Glioma cells [73]. The electrospun nanofibers for drug delivery systems are generally produced either by blend electrospinning [60], or by coaxial electrospinning methods [6, 7, 68, 74]. In blend electrospinning technique, the drug is mixed with polymer solution prior to electrospinning process, whereas in coaxial electrospun technique [6, 7], the nanofibers are produced with core-sheath structures having drug distribution in the core of the fibers [66, 67]. The electrospun nanofibrous scaffolds have been fabricated successfully by blending drugs in polymer solution before electrospinning fibers. Lu et al. [75] have prepared successfully, the electrospun nanofibers for sustained release of plasmid DNA using synthetic biodegradable polymers, such as PLA, poly(glycolic acid) (PGA), and amphiphilic block copolymers of PLA and poly(ethylene glycol) (PEG). Since plasmid DNA was not compatible with studied polymer systems; hence plasmid DNA was localized more at the surface of nanofibers and shown enhanced bulk release of plasmid DNA from electrospun nanofibrous scaffolds.

The coaxial electrospinning technique is found to be more useful in loading of various drugs [6, 7], and bioactive agents in polymers [76], which are forced through a coaxial capillary channel for electrospinning in presence of applied potential. Investigations have indicated that the coaxial technique of drug delivery systems is particularly useful to protect the drugs, which are easily denatured or decomposed during electrospinning of nanofibrous scaffolds. Therefore, coaxial electrospinning technique has been used successfully in fabrication of nanofibrous scaffolds loaded with liposomes without decomposition and denaturization [77]. The loading and drug release in electrospun nanofibrous scaffolds was found to be dependent on molecular weight of the drugs, and drug release behavior has varied with the increase in molecular weight of loaded drugs in poly(vinyl alcohol) (PVA) based electrospun nanofibrous scaffolds [78]. The bulk release problem of electrospun nanofibers has been controlled using cross-linkers, which controlled the diffusivity of encapsulated drugs from the nanofibrous scaffolds. The gelatin cross-linked poly(lactic acid-co-glycolic acid) (PLGA) nanofibers based scaffolds have shown significant reduction in the bulk release of encapsulated fenbufen [79, 80], and similar trends are found with PVA cross-linked electrospun nanofibrous scaffolds [72]. Although in the beginning, it was thought that electrospinning causes the denature of loaded drug, subsequent studies have clearly demonstrated that the condition of electrospinning could be carefully tuned to maintain the chemical integrity of loaded drug [78]. The PLGA based electrospun nanofibrous scaffolds have been used successfully in sustained release of anti-cancer drug for *in vitro* treatment of C6 Glioma cells [79]. The differential scanning calorimetric (DSC) studies have indicated that loaded drugs are able to form solid solution in the polymers by reducing drug-drug interactions [3–5]. The electrospun nanofibrous scaffolds have shown a sustained release of paclitaxel for more than 60 days comparable to commercially paclitaxel formulation-Taxol [79]. The water soluble polymers have been used successfully in the fabrication of electrospun nanofibrous scaffolds for the delivery of water insoluble drugs, such as intraconazole, which found to be dispersed homogeneously in amorphous polymers [62]. Zeng et al. [58] have used PLLA containing rifamin and paclitaxel to produce uniform ultrafine nanofibrous scaffolds for the treatment of tuberculosis and cancer. The analysis of nanofibers by optical and electron microscopy has precluded the formation of rifamin crystals at the surface of nanofibers and drug release found to be constant in presence of enzyme. In these investigations, the bulk release was absent, which was a clear indication for the homogeneous distribution of loaded drug in the nanofibers, and drug release has mainly taken placed through enzymatic degradation of PLLA and insignificant drug release has taken place through diffusion or solvent penetration in the nanofibers. Since rifamin and paclitaxel are the lipophilic drugs; hence they were solubilized easily in PLLA but doxorubdin hydrochloride found to be on the surface of the nanofibers as it was not soluble in the nanofibers, which clearly indicated that the solubility of drugs has played a significant role in drug distribution and release profile from the electrospun nanofibrous scaffolds. The technique of coaxial electrospinning has been used for the delivery of plasmid DNA and non-viral gene system, and the monitoring of gene delivery vector for about 60 days found to be consistant [68]. The properties of gene delivery vector have been controlled by using different amount of vector and core polymers of different molecular weights. The core-sheath electrospun nanofibrous scaffolds have shown great potential in the delivery of bovine serum protein from poly(ε-caprolactam) (PCL) based nanofibrous scaffolds. The protein was distributed homogeneously in the cores of the fibers; hence nanofibrous scaffolds have shown better release of loaded protein [68]. The water in oil emulsion has been used to fabricate coaxial electrospun nanofibers. The aqueous phase was having poly(ethylene oxide) (PEO) and chloroform was containing amphiphilic poly(ethylene oxide)-poly(L-lactic acid) diblock copolymer [80]. The coaxial electrospun nanofibers for the delivery of protein containing PEO in the core has protected 75% of initial activity of the protein [69]. The coaxial electrospun nanofibers have shown better control on the bulk release of core encapsulated growth factor, which is reduced to 17.4% in comparison to blended nanofibers, which shown a bulk release of 43.8% within initial period of 6 h [72]. Further control in initial bulk release of drugs from electrospun PCL nanofibers is made using 0–50 wt% of poly(glycerol monostearate-co-ε-caprolactone) (PGC-C18) hydrophobic agent, which has reduced initial burst release and prolonged the sustain release of encapsulated model bioactive agent SN-38 (7-ethyl-10-hydroxycampthothecin) [81]. The addition of PGC-C18 hydrophobic agent in PCL electrospun nanofibers has shown a maximum bulk release of 10% of campthothecin-11 (CPT-11) within a period of 9 weeks but without PGC-C18 hydrophobic agent, the initial bulk release was 60% within a few days [82]. The hydrophobicity of electrospun nanofibers has been controlled by the entrapment of air, which prevented the penetration of water and hydrolysis of polymer; hence nanofibers were able to show reduced initial bulk release and prolonged sustained release of loaded drugs. However, air trapped electrospun nanofibers have shown a poor distribution of the drugs. To overcome the problem of drug interactions and drug release from the electrospun nanofibers loaded with dual or multiple drugs, the fabrication of particle/polymer electrospun composites in the drug delivery system is becoming a topic of current interest [80, 83–85]. In this technique, the nanoparticles prepared separately have been used in emulsion electrospining [81–84]. To prevent the interactions with hydrophobic rhodamine-B (RHB) and hydrophilic fluorescein (FLU) in PLGA electrospun fibers, the mesoporous silica nanoparticles have been loaded together with a solution containing PLGA and drugs before electrospinning. The particles were distributed homogeneously and were successful in preventing the interactions between RHB and FLU [81]. To prevent the fast release of FLU from nanofibers, the RHB and FLU were loaded separately on mesoporous silica particles before mixing with PLGA solution and electrospinning the nanofibers. This strategy has reduced the released rate of FLU from the nanofibers, and has shown prolonged release of FLU [82]. The cellulose acetate phthalate (CAP) based electrospun nanofibers containing antiviral drugs have been electrospun successfully to inhibit HIV infection from man to women during sexual intercourse without impeding the proliferation of vaginal microflora [86]. Currently the colon drug delivery system has attracted the attention of people both in pharmaceutical industry and academia to develop a delivery system for the treatment of diseases associated with colon such as colon cancer, ulcerative colitis and diarrhea. Colon is a suitable site for the delivery of poorly adsorbed drugs due to its long retention time. The nanofibers based colon delivery systems was found to be useful substitutes for large delivery systems and electrospun nanofibers loaded with mixture of ERS and ES in ethanol have been fabricated containing colon specific indomethacin [87]. To ascertain the homogeneous distribution of indomethacin in the nanofibers, the nanofibers were analyzed by differential scanning calorimetry [3–6]. The absence of drug-polymer interactions has been supported by FT-IR analysis. The nanofibers were electrospun using appropriate polymer-drug ratios and optimizing the viscosity of polymer solution and type of solvents. The drug release profile of diclofenac sodium from electrospun nanofibers found to be pH dependent and exhibited better drug release in colon than that of a physical mixture of diclofenac sodium and Eudragit L 100–55 polymer [88]. Although electrospun nanofibers are excellent drug carriers but there are still some issues that need to be addressed. One challenge is to control the bulk release of drugs that arises due to surface adsorption and aggregation of drugs at the surface of electrospun fibers [89], especially when drug loading is higher. However, coaxial and emulsion methods of electrospinning have produced nanofibers with significant reduction in initial bulk release [90, 91]. Other challenges are to retain the bioactivity or functional efficiency of loaded drugs in the fibers on application of high voltage and ultrasonication during blending of drugs in the polymer solution [92]. The distribution of loaded drugs has also shown significant influence on their activities in the nanofibers such as surface deposition of DNA in chitosan nanofibers has shown different activity then its homogeneous distribution in the fibers [93].

### Electrospun nanofibrous scaffolds in wound healings

The polymer-based of electrospun nanofibrous scaffolds play significant role in wound healings; hence, selection of appropriate polymer is very important to obtain nanofibrous scaffolds that would match the required properties of wound healing materials. Polysaccharides such as chitosan, cellulose, and hyaluronic acid (HA) as well as proteins, such as collagen and silk have been electrospun for localized drug delivery applications [94]. Many of these polymers have specific properties that promote wound healing. For instance, chitosan exhibits both antibacterial and hemostatic activities. The HA has been used as biomaterial in various fields of biomedical research such as, dermal filler scaffolds for tissue engineering and drug delivery devices [95]. The physical and biological activities of 1,4-butanedioldiglycidyl ether cross-linked HA has been evaluated recently, which clearly indicated that HA particles were having excellent biocompatibility; hence might be useful in fabrication of wound healing nanofibrous scaffolds. Similarly recent studies on carboxymethyl cellulose (CMC) hydrogels have indicated that CMC gels could be used in drug delivery systems and wound healing applications [96]. Synthetic polymers which are commonly used for wound healing applications include PEO [97–99], PLA [75, 100–103], PCL [104], and PVA [105]. Electrospun nanofibrous scaffolds of these polymers have displayed high mechanical strength in comparison to natural polymers. Additionally, synthetic polymers were found to be soluble in a wider range of solvents, which facilitated their electrospinning process [106]. Often wound healing scaffolds from biopolymers are electrospun in conjunction with a synthetic polymer in order to fine-tune the mechanical, degradation, and/or morphological features of the porous membranes to accomplish the needs of individual patient. To accelerate the wound healings and to reduce the postsurgical infections, the release of two or more different drugs at proper time and appropriate doses, has been achieved with the help of electrospun fibers [107, 108]. After surgical operations, usually the infection and pain occurs frequently; hence, sustained release of antibiotics and analgesics from electrospun fibers has been found useful to overcome the post-surgical complications. The PLLA, poly(orthoester) (POE), and PLGA [108] nanofibers loaded with drugs have been used in sustained and controlled release applications. The implants used in wound dressings must have optimal mechanical strength to support the healing process [109]. The poly(ethylene-co-vinyl alcohol) (EVOH) electrospun nanofibrous scaffolds loaded with Ag nanoparticles have been used as dressing material in skin wound healings to prevent the inflammation by controlled release of Ag nanoparticles [110]. The nanofibers based dressing materials have shown better clinical properties in skin wound treatment as compared to existing woven and nonwoven materials. The anti-bacterial properties of electrospun nanofibers have been evaluated with staphylococcus aureus, which is one of the main pathogenic bacteria found on the surfaces of skin burns. To assess the pathogen-restraining ability of Ag encapsulating naofibers four samples with different amount of Ag nanoparticles were prepared ranging from 0.03-0.15 g in 10 mL 80:20 mixture of 2-propanol and water. A linearly increasing effectiveness is found with highest Ag concentration yielding in the biggest loop for the same area of the fiber patches, clearly indicated a stronger pathogen-restraining effect but no bacteriostatic loop was observed for pure EVOH fibers without Ag.

### Electrospun nanofibrous scaffolds in delivery of biogenic molecules

The development of tissue-engineered organs requires the maintenance of cell viability and differentiated function, encouragement of cell adhesion, cell proliferation, and modulation of direction and speed of the cell migration. The cell activities are controlled by the delivery of various growth factors such as, transforming growth factor-β1 (TGF-β1) to induce osteogenesis and chondrogenesis from bone marrow–derived mesenchymal stem cells. Also, brain derived neurotrophic factor (BDNF) is able to enhance regeneration of spinal cord injury. The presence of hydroxybutylate or β-mercaptoethanol can help the differentiation of bone marrow-derived mesechymal stem cells to neuronal cells. The release of these biomolecules from electrospun nanofibrous scaffolds is useful to control and regeneration of diseased organs. The easiest method for the delivery of bioactive molecules is the injection near the site of proliferating and differentiating cells, but direct injection of biomolecules is difficult due to their short life time, high molecular weight and size of the growth factors. The growth factors are soluble signaling proteins capable of instructing specific cellular responses for cell proliferation, migration and differentiation [111]. However, due to short biological stability, the direct delivery to the site is difficult. The fabrication of nanofibrous scaffolds loaded with growth factors is found to be an useful strategy for control release of growth factors in biological systems. The growth factors have low tissue penetration and their toxicity is another problem, which needs to be considered during their applications [112]. Therefore, locally controlled release of bioactive molecules is possible by impregnating fibrous scaffolds, which would control the structures and release profile of bioactive molecules. The activity of bioactive molecules depends on the type of material and methods of their conjugation with scaffolds. The tethering of insulin and transferring on the surface of poly(methyl methacrylate) has shown different activity in the growth of fibroblast as compared to dispersed or physically adsorbed molecules of the protein [113]. Electrospun nanofibers have been found useful in site specific delivery of various biogenic molecules and for the treatment of various infections and cancers. The loaded biogenic molecules are released in therapeutic dosage from the electrospun nanofibrous scaffolds. The inherent high porosity of electrospun nanofibers is responsible for their precise degradation and release of loaded drugs. PLGA and PLA–PEG block copolymer-based nanofibrous scaffolds have been used successfully for the delivery of plasmid [114]. The delivery of biogenic molecules can be controlled by tuning the composition of fibers and their morphology. The angiogenic factors, such as androgen play critical roles in proliferation and migration of keratinocytes, fibroblasts, and endothelial cells to promote angiogenesis and wound healings [115]. The delivery of growth factors by electrospun nanofibers remains a challenge as the growth factors could lose their bioactivity during the harsh process of nanofiber formation [116]. The collagen-PCL electrospun nanofibers loaded with synthetic anti-androgen receptor [5-hydroxy-1,7-bis (3,4-dimethoxyphenyl)-1, 4, 6-heptatrien-3-one] have been fabricated and evaluated for their activity [117]. The bioactive molecules loaded nanofibers have facilitated, cells migration, cells growth, and differentiation in wound healing and skin regeneration.

### Electrospun nanofibrous scaffolds in delivery of catalysts

The polystyrene electrospun nanofibrous scaffolds have been used for the delivery of α-chymotripsin and catalytic activity in biotransformation [118]. The nanofibrous delivery of α-chymotripsin has shown enhanced hydrolytic activity (65%) in comparison to direct applications. The PVA electrospun nanofibers have been used to study the releases kinetics of protein and its bioactivity in physiological conditions [119]. The nanofibrous scaffolds were able to release protein in a controlled and sustained manner without altering the bioactivity of released protein.

### Electrospun nanofibrous scaffolds in tissue engineering

**Figure 2:**
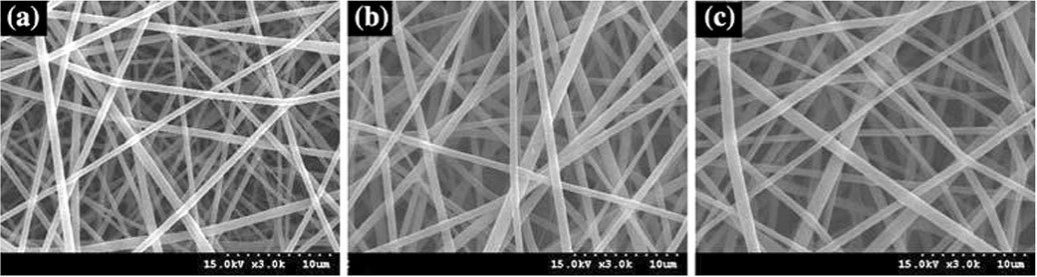
**SEM micrographs of electrospun nanofibrous scaffolds using a PHBV/gelatin solution at TFE 6 wt%; (a) 30/70, (b) 50/50, and (c) 70/30 (adapted from reference** [127] **).**

**Figure 3:**
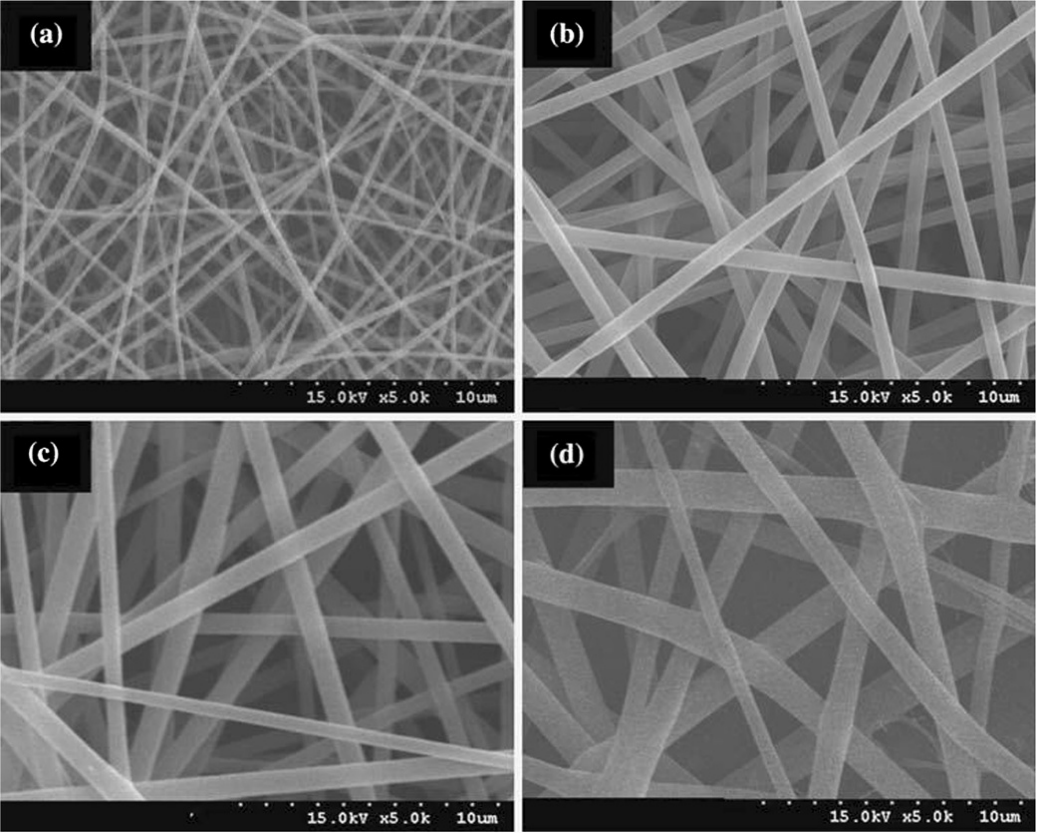
**SEM micrographs of electrospun nanofibrous scaffolds using TFE solutions with different amount of PHBV/gelatin (50/50); (a) 2 wt%, (b) 4 wt%, (c) 6 wt%, and (d) 8 wt% (adapted from reference** [127] **).**

Amongst the various types of scaffolds, the electrospun nanofibrous scaffolds have attracted great attention in tissue engineering due to high surface to volume ratio and due to various possibilities to control their properties and applications [120–123]. The electrospun nanofibrous scaffolds are cost effective and able to act as extra cellular material to provide cell adhesion, proliferation and differentiation. The electrospun nanofibers for bone tissue culturing and regeneration utilize a range of biopolymers with synthetic origin, which include poly(α-hydroxyl acid) and poly(hydroxyalkanoate), such as poly(hydroxybutyrate) (PHB). The natural polymers such as collagen, gelatin, silk, and chitosan are also considered useful for bone tissue regeneration. The PCL has long been studied as a degradable nanofiber matrix for bone regeneration [124] and Rat bone marrow has demonstrated the secretion of type I collagen and calcium mineralization within 4 weeks of culturing. The PLA nanofibers have also shown good response toward MC3T3-E1 bone cells [125]. The PHB and poly(hydroxybutyrate-co-hydroxyvalerate) (PHBV) electrospun nanofibers have shown enhanced osteoblastic activity equivalent to flat membrane scaffolds [126]. The gelatin has been used as a tissue engineering material by blending with PHBV, due to its low cost and hydrophilicity. The PHBV/gelatin nanofibrous scaffolds were obtained by co-electrospinning a transparent polymer solution of gelatin and PHBV in 2,2,2-trifluoroethanol (TFE) at a volume ratio of 50/50 [127]. The effect of gelatin on the morphology of nanofibrous scaffolds was examined using different ratios of PHBV and gelatin (Figure 2).A smooth and uniform electrospun nanofibrous scaffolds were obtained by varying the weight percent from 2-8% in the solvent (Figure 3).

The PHBV and collagen (Col) nanocomposite in 1,1,1,3,3,3-hexafluoro-2-isopropanol (HFIP) has also been used successfully for electrospinning of nanofibers for tissue engineering [128] at different weight ratios of PHBV and collagen and using spinning parameters as shown in Table 1.

**Table 1:** **Electrospinning conditions for various amounts of polymer solution**

	Concentration (wt%)	Voltage (kV)	Distance* (cm)	Flow rate (mL/h)
PHBV	2	7	15	1.5
PHBV-Col	2	12	22	1.0
Collagen	2	20	15	1.5

*Distance is from the spinneret to grounded collector (adapted from reference [128]).

**Figure 4:**
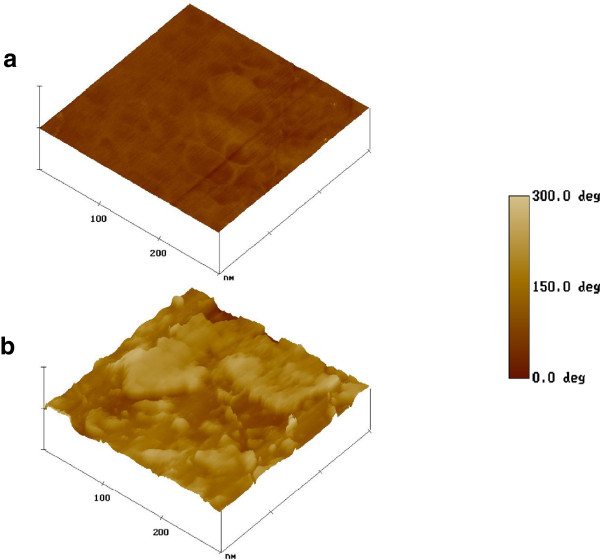
**AFM phase images of nanofibrous scaffold surface; (a) PHBV and (b) PHBV–Col (Adapted from reference** [128] **).**

On acceleration of solution jet toward grounded collector, the solvent evaporated and a charged polymer nanofiber was deposited on grounded collector as nanofiber web. The electrospinning of nanofibrous scaffolds was done at different weight ratios of PHBV and collagen (7:3, 5:5, and 3:7), which produced nanofibrous scaffolds with smooth morphology. The addition of PHBV in collagen has produced nanofibrous scaffolds with heterogeneous surfaces, which enhanced the application of electrospun nanofibers in tissue engineering (Figure 4). The synthetic polymers after blending with natural polymers have been used successfully in tissue engineering. The electrospun nanofibrous scaffolds obtained from the gelatin blends with PCL and PLA have shown better proliferation and expression for osteoblastic cells in comparison to pure PCL and PLA [129, 130]. The blending of collagen with PCL has proved to be useful in improving the mechanical properties of electrospun fibers as percent elongation has increased significantly on addition of collagen in PCL without altering the tensile strength of original PCL [131].

The electrospun nanofibrous scaffolds fabricated from PEO blended silk and loaded with BMP-2 have shown higher osteogenic differentiation and calcification as compared to nanofibrous scaffolds obtained without BMP–2 [132].

### Electrospun nanofibrous scaffolds in vascular graft applications

The electrospinning technique has also been found useful in fabrication of nanofibrous scaffolds for cardiovascular tissue engineering and small-caliber vascular grafts using solution of PLA or PCL in methylene chloride. The solution of PLA or PCL in methylene chloride was loaded into 1 mL syringe containing blunt 18 gauge needle at a voltage ranging from 10–15 kV and circular cylindrical counter electrode was used to produce tube like structures. A tube like vascular graft has been also been developed using solution of PLA and water soluble elastin in presence of triethylamine [133]. These investigations have clearly indicated that electrospinning could also be used in fabrication of vascular grafts and scaffolds for biomedical applications.

## Conclusions

The various studies reported on applications of nanofibrous scaffolds have clearly indicated that the technique of electrospinning is of great significance and useful in fabrication of nanofibrous scaffolds for biomedical applications using biodegradable synthetic and natural polymers. Though the technique of electrospinning is quite old but it is able to produce micro to nanometer sized fibers for tissue engineering, delivery of regenerative medicines and wound healing applications. The onsite delivery of catalysts and bioactive molecules could be achieved with the help of electrospun nanofibers without any loss in their activities and structures. The electrospinning is emerging as an interdisciplinary area of research and possess tremendous scope for its improvements by using suitable biomaterials and controlling fabrication parameters. The techniques of coaxial and emulsion electrospinning are the areas of current interest and could be used in fabrication of drug loaded nanofibers for biomedical applications. Tissue engineering is an interdisciplinary approach for tissue regeneration through integration of specific cells with electrospun nanofibers. Similarly wound healing using nanofibrous scaffolds is an ideal therapeutic option for the treatment of the burns and defected tissues. The electrospun nanofibrous scaffolds could be a suitable substitute for invasive bone transplantation by modifying their properties with biomolecules and bone morphogenic proteins like BMP-2.

## Abbreviations

PLA: Poly (lactic acid)
PVP: Poly (vinyl pyrrolidone)
PEVA: Poly (ethylene-co-vinyl acetate)
PGA: Poly (glycolic acid)
PEG: Poly (ethylene glycol)
PVA: Poly (vinyl alcohol)
PLGA: Poly (lactic acid-co-glycolic acid)
PLLA: Poly (L-lactic acid)
PCL: Poly (ε-caprolactam)
PEO: Poly (ethylene oxide)
PGC-C18: Poly (glycerol monostearate-co-ε-caprolactone)
CMC: Carboxymethyl cellulose
PHB: Poly (hydroxybutyrate)
PHVB: Poly (hydroxybutyrate-co-hydroxyvalerate).
